# Enhancing Military Women's Health and Readiness Through Targeted Research Initiatives

**Published:** 2025-05-01

**Authors:** Michelle D. Lane, Tracy L. Behrsing, Miriam E. Redington, Akua N. Roach, Dwayne L. Taliaferro, Katharine W. Nassauer, Gayle Vaday

**Affiliations:** Congressionally Directed Medical Research Programs, U.S. Army Medical Research and Development Command, Fort Detrick, MD

Health and wellness are vital aspects of military mission readiness. The Congressionally Directed Medical Research Programs (CDMRP), a funding organization within the Defense Health Agency (DHA), supports research on over 90 different diseases, conditions, and topics that are directed by Congress. The CDM-RP's vision is to transform health care through innovative and impactful research.

The CDMRP has implemented multiple initiatives to enhance women's health since its inception in 1992. Military women's health care needs and experiences differ from those of male service members, in addition to those of civilian women. Women who served in the military have a higher risk or incidence of some conditions compared to the civilian population, including breast cancer, ^1^ multiple sclerosis, ^2^ dementia, ^3^ and eating disorders. ^4^ Better prevention, diagnosis, and treatment ensure mission readiness not only as it relates to the service members' health, but for the health and well-being of their families as well. ^5^

To achieve its vision, the CDMRP adheres to a mission of responsibly managing collaborative research that discovers, develops, and delivers health care solutions for service members, veterans, their families, and the American public. In fiscal years 2022-2023, the CDMRP funded approximately $979.1 million in women's health research across many different conditions and topics that affect women exclusively, disproportionately, or differently **(Table)** .

**TABLE.** CDMRP Investments in Women's Health Research, Fiscal Years 2022–2023

Research Topic	Investment (millions)	Number of Awards
Breast cancer	$264.8	159
Neurological disorders	$178.6	140
Psychological health	$106.9	49
Gynecological cancers	$103.3	128
Other ^a^	$94.1	56
Autoimmune disorders	$67.1	73
Other cancers	$50.9	65
Cardiovascular diseases	$37.7	17
Alzheimer's disease	$26.3	23
Pain	$20.2	15
Musculoskeletal disorders	$13.7	11
Sensory system disorders	$8.8	10
Gynecological and reproductive disorders	$6.5	9
Grand Total	$979.1	755

a Includes diabetes, fragile X syndrome, Gulf war illness, neurofibromatosis, pancreatitis, Rett syndrome, toxic exposures, tuberous sclerosis complex.

This editorial provides an overview of the CDMRP and its women's health research initiatives, including the directive on Inclusion of Women and Minorities as Subjects in Clinical Research and the directive on Sex as a Biological Variable in Research. The editorial also describes efforts to prioritize women's health research and high-lights examples of relevant CDMRP-funded studies to advance military women's health research.

## CDMRP Women's Health Initiatives—Past to Present

Women's health is a foundational element of the CDMRP. The breast and ovarian cancer research programs established by the U.S. Congress in 1993 and 1997, respectively, provided funding that contributed to U.S. Food and Drug Administration (FDA)-approved drugs in addition to improved diagnostic approaches, prognostic tests, and changes in clinical practice. Those advancements continue to benefit service members and beneficiaries throughout the Military Health System (MHS).

Over the past 30 years, women's health research funded by the CDMRP has expanded to include Alzheimer's disease, autoimmune disorders (e.g., lupus, rheumatoid arthritis, scleroderma, celiac disease), cardiovascular disease, endometriosis, various cancers, sleep disorders, and chronic fatigue syndrome. Patients, survivors, and caregivers—including active duty service members and veterans—add critical perspectives and a sense of urgency within every aspect of the CDMRP's program cycle, serving as full voting members on both peer review panels as well as programmatic panels that contribute to funding decisions, program priorities, and investment strategies.

Consistent with *The Belmont Report* ^6^ and Congressional legislation, inclusion of women and minorities in clinical studies funded or supported by the CDMRP has been emphasized. Since 2009, the CDMRP's funding opportunity announcements have contained language encouraging the inclusion of women and minorities in clinical trials so the burdens and benefits of participating in clinical research may be applied to all affected populations. The CDMRP collaborated with partners at the National Institutes of Health (NIH) Office of Research on Women's Health, a leader in women's health research initiatives, to develop 2 important directives to ensure results from CDMRP-funded studies benefit both men and women from affected populations. These directives mirror policies already established by the NIH ^9 , 10^ and incorporate their many years of lessons learned.

The first directive, in 2020, requires inclusion of women and minorities, as appropriate for a study's objectives, in all clinical research studies funded by the CDMRP, including clinical trials. ^7^

In 2024, the CDMRP took its women's health initiatives a step further by implementing its directive requiring researchers to consider sex as a biological variable in their research designs and reporting, including pre-clinical studies with animal models. ^8^ Failure to account for sex as a biological variable may undermine research rigor, transparency in participant population selection, and generalizability of research findings.

In fiscal year 2024 funding opportunities, the CDMRP began explicitly encouraging research on health areas and conditions that affect women uniquely, disproportionately, or differently from men. This research should relate anticipated findings to improvements in women's health outcomes or advancements in knowledge for women's health. Several programs have made targeted efforts to prioritize women's health as a focus area. The Peer Reviewed Orthopaedic Research Program, for example, offered a funding mechanism specifically to support research on orthopaedic injuries affecting women in combat roles.

Groundbreaking research is underway to advance military women's health throughout the many programs managed by the CDMRP. Three current CDMRP-funded studies advancing research for prevention and treatment of important conditions—breast cancer, pelvic pain, and post-traumatic stress disorder (PTSD)—with unique effects on military women are described in the following section.

## CDMRP-funded Study Highlights

### Breast Cancer Risk and Polycyclic Aromatic Hydrocarbons

Breast cancer is the most common non-skin cancer in women and the deadliest cancer in women under age 40 years, ^11 , 12^ and its incidence rate in women aged 40-59 years is higher among active duty service members than in the general population. In 2020, the CDMRP's Breast Cancer Research Program funded a research project totaling $3.1 million to address the challenge of identifying determinants of breast cancer initiation, risk, and susceptibility among female active duty service members. Cumulative environmental exposures have been identified as a potential factor that may contribute to the difference in breast cancer rates among women of similar ages in military service and the civilian population. ^1^

Investigators Celia Byrne, PhD, at the Uniformed Services University of the Health Sciences, and Mary Beth Terry, PhD, at Columbia University Medical Center, are partnering in a case-control study utilizing the Department of Defense (DOD) Cancer Registry in addition to serum samples and data from the DOD's Armed Forces Health Surveillance Division (AFHSD). These investigators aim to quantify exposure to environmental contaminants called polycyclic aromatic hydrocarbons and evaluate breast cancer risk associated with exposures to such contaminants for female active duty service members. The study will also evaluate whether genetic variations affect susceptibility to environmental exposures.

If successful, findings from this study will identify specific exposures that contribute to breast cancer risk, leading to further understanding of the biological factors involved in breast cancer incidence.

### Pelvic Pain Conservative Care in Female Service Members

Chronic pelvic pain, defined as persistent or recurrent pain perceived to be in and around the pelvis that lasts for at least 6 months that is not attributable to cancer, ^13 , 14^ disproportionately affects female service members. The disproportionate occurrence of chronic pelvic pain in female service members may result from higher incidence of hip and pelvic injuries, inadequate urogenital hygiene in deployed environments, or sexual trauma. ^15 , 16^

The CDMRP's Peer Reviewed Orthopaedic Research Program funded a $2.5 million research project in 2024 to assess clinical effectiveness and physiological efficacy of 3 physical therapy treatments for chronic pelvic pain in female service members. This clinical trial, led by Shane Koppenhaver, PhD, at Baylor University, will randomly assign 300 women with chronic pelvic pain to 3 treatment groups. The study will test the hypothesis that field-expedient interventions are superior to usual care and not inferior to the highest or ‘gold standard’ of care. These interventions could enable injured female service members to remain on the battlefield or on mission without need for evacuation or medical discharge.

In addition to potential changes in clinical care guidelines, the study team aims to develop a clinical decision tool to predict subgroups of women with chronic pelvic pain who are most likely to benefit from emerging field-expedient care and differentiate those requiring ‘gold standard’ intravaginal specialist care.

### Post-Traumatic Stress Disorder Prevention and Treatment with Microbiome-based Therapies

Women, service members, and veterans are more likely to develop PTSD than the general population, and there is an urgent need to identify more effective interventions. ^17 , 18^ To address this need, in 2023 the CDMRP's Traumatic Brain Injury and Psychological Health Research Program awarded $6.3 million to Brigham and Women's Hospital, for Yang-Yu Liu, PhD, to examine and target the relationship between PTSD and the gut microbiome, employing both human and animal studies.

Dr. Liu aims to develop a synbiotic therapeutic of prebiotics and probiotics to reduce PTSD symptoms. The study utilizes existing data and resources from the large, well-characterized cohort of women in the Nurses' Health Study II. ^19^ This CDMRP-funded study's 4 research projects will 1) differentiate the gut microbiomes in women who have experienced PTSD, trauma, or resilience, 2) reveal causation between PTSD and gut microbiome using a novel computational method, 3) quantify the effect of trauma exposure on the gut microbiome and neuronal activity in mice, and 4) test the efficacy of microbiome-based therapeutics in reversing stress-induced behavioral alterations in mice.

If successful, this effort will provide foundational evidence for microbiome-targeted interventions for PTSD treatment for improved life quality and function among women and others affected by PTSD.

## Editorial Comment

Many more CDMRP-funded women's health-related studies are complete or ongoing. Results from these funded studies will continue to advance the medical profession's understanding and ability to prevent, assess, and treat diseases and conditions that affect military women's readiness. Prioritizing research on the diseases and conditions that affect women uniquely, disproportionately, or differently, considering sex as a biological variable in all stages of research while ensuring appropriate representation of the diverse affected populations in clinical research, will accelerate medical solutions for all military women and enhance mission readiness. The CDMRP's ongoing commitment to women's health research will contribute to research advancements that will benefit not only service members, but their families, veterans, and the American public at large.

To learn more about the CDMRP's programs and funded studies, or receive funding opportunity notifications by email, visit https://cdmrp.health.mil . Women's health portfolio inquiries can be submitted to dha.detrick.cdmrp.mbx.public-affairs@health.mil .
